# Development of Liposomal Vesicles for Osimertinib Delivery to EGFR Mutation—Positive Lung Cancer Cells

**DOI:** 10.3390/pharmaceutics12100939

**Published:** 2020-09-30

**Authors:** Paulina Skupin-Mrugalska, Tamara Minko

**Affiliations:** 1 Department of Inorganic & Analytical Chemistry, Poznan University of Medical Sciences, Grunwaldzka 6, 60-780 Poznan, Poland; 2 Department of Pharmaceutics, Ernest Mario School of Pharmacy, Rutgers: The State University of New Jersey, 160 Frelinghuysen Road, Piscataway, NJ 08854, USA; minko@pharmacy.rutgers.edu; 3 Rutgers Cancer Institute, Rutgers, the State University of New Jersey, 195 Little Albany Street, New Brunswick, NJ 08903, USA; 4 Environmental and Occupational Health Science Institute, Rutgers, the State University of New Jersey, 170 Frelinghuysen Rd., Piscataway, NJ 08854, USA

**Keywords:** liposomes, drug delivery, tyrosine kinase inhibitors, osimertinib, non-small cell lung cancer, EGFR resistance mutations

## Abstract

Osimertinib (OSI, AZD9291), is a third-generation, irreversible tyrosine kinase inhibitor (TKI) of the epidermal growth factor receptor (EGFR) that selectively inhibits both EGFR-TKI–sensitizing and EGFR T790M resistance mutations. OSI has been approved as a first-line treatment of EGFR-mutant lung cancer and for metastatic EGFR T790M-mutant non-small cell lung cancer. Liposome-based delivery of OSI can provide a new formulation of the drug that can be administered via alternative delivery routes (intravenous, inhalation). In this manuscript, we report for the first time development and characterization of liposomal OSI formulations with diameters of ca. 115 nm. Vesicles were composed of phosphatidylcholines with various saturation and carbon chain lengths, cholesterol and pegylated phosphoethanolamine. Liposomes were loaded with OSI passively, resulting in a drug being dissolved in the phospholipid matrix or actively via remote-loading leading to the formation of OSI precipitate in the liposomal core. Remotely loaded liposomes were characterized by nearly 100% entrapment efficacy and represent a depot of OSI. Passively-loaded vesicles released OSI following the Peppas-Sahlin model, in a mechanism combining drug diffusion and liposome relaxation. OSI-loaded liposomes composed of l-α-phosphatidylcholine (egg-PC) demonstrated a higher toxicity in non-small lung cancer cells with EGFR T790M resistance mutation (H-1975) when compared with free OSI. Developed OSI formulations did not show antiproliferative activity in vitro in healthy lung epithelial cells (MRC-5) without the EGFR mutation.

## 1. Introduction

For several decades, lung cancer has been the most common cancer worldwide and has been accounted for the most common cause of cancer-related deaths [1,2]. Among all types of lung cancer, non-small cell lung cancer (NSCLC) represents approximately 85% of lung cancer cases with a lower than 15% 5-year survival [1,3]. Genetic and molecular profiling of NSCLC has led to the discovery of molecular alterations that drive tumor initiation and progression [4]. The above findings have revolutionized treatment management in NSCLC and have helped to improve treatment outcomes and quality of life compared to traditional chemotherapy [5]. In NSCLC, several targetable significant pathways have been identified, including EGFR, PI3K/AKT/mTOR, RAS–MAPK, and NTRK/ROS1 pathways [5,6]. EGFR mutations were the first molecular alterations in NSCLC, discovered in 2004 [7], and they occur in 10–28% of NSCLC patients [8]. In response to the discoveries at a molecular level, drugs targeting the EGFR pathway, tyrosine kinase inhibitors (TKIs) have been developed and shown clinical benefits. First-generation (gefitinib and erlotinib) and second-generation (afatinib) TKIs demonstrated superior progression-free survival, objective response rate, and quality of life compared to standard chemotherapy in patients whose tumors harbored canonical activating EGFR mutations [9,10,11]. Unfortunately, more than 60% of patients develop resistance to first- or second-generation TKIs. The common mechanism of acquired resistance to EGFR TKIs is the EGFR T790M mutation [12]. Third-generation inhibitors were created to target the T790M mutations while maintaining activity against the original exon19del and L858R mutations [6].

Osimertinib, (OSI, AZD9291) is a third-generation, irreversible TKI of the epidermal growth factor receptor (EGFR-TKI) that selectively inhibits both EGFR-TKI–sensitizing and EGFR T790M resistance mutations [13]. OSI initially gained FDA approval for patients with metastatic EGFR T790M-mutant NSCLC that had progressed on first- or second-generation EGFR TKIs and has subsequently achieved approval as a first-line treatment of EGFR-mutant lung cancer [6,13,14]. OSI is administered orally in the form of film-coated tablets (TAGRISSO^®^) containing OSI as mesylate. The absolute bioavailability of OSI was determined as 70% [15].

Several drug delivery strategies have been previously proposed to enhance the safety and efficacy of TKI-mediated therapy in lung cancer [16,17,18,19,20,21]. Liposomes are predominant nanocarriers among these delivery systems that have been developed for TKIs delivery. The high interest in liposomes as a TKIs delivery strategy results from its biocompatibility, high encapsulation of lipophilic drugs with the ability to load hydrophilic cargo and surface modifications, e.g., with PEG molecules and targeting moieties [22]. Liposomes are widely accepted as a drug delivery system by regulatory agencies and used as delivery agents in many approved medicinal products [23].

OSI has an acceptable bioavailability after oral administration. However, it has a high in vitro plasma protein binding of 94.7%. OSI has been demonstrated to bind covalently to rat and human plasma proteins, human serum albumin and rat and human hepatocytes [15]. The high affinity for endogenous proteins may raise the risk of adverse drug reactions and decrease the safety profile of the drug [21]. Therefore, we hypothesized that a drug delivery approach combined with local pulmonary delivery could be an attractive alternative to improve the efficacy and safety of lung cancer TKI-based targeted therapy [24,25]. Liposome encapsulation can result in increased accumulation of the drug in the tumor due to the enhanced permeability and retention (EPR) effect [26], leading to more selective deposition of EGFR inhibitor and reduced impacts on critical healthy tissues. Liposome-based delivery of OSI can provide a new formulation of the drug that can be administered by different routes (intravenous, inhalation), especially in patients who cannot take medicines orally and with advanced NSCLC. Such delivery can potentially enhance the efficacy of the treatment and decrease toxicity of drug(s) [24,27,28,29]. In patients with EGFR mutation-positive advanced NSCLC, there is an unmet need for EGFR-TKIs with improved central nervous system (CNS) penetration and activity against CNS metastases, either at initial diagnosis or time of progression [30]. OSI was shown to have a good brain exposure in human subjects [31] and to be active against CNS brain metastases or leptomeningeal disease, regardless of T790M status [30,32,33]. Patients with brain metastasis may benefit from treatment with liposome-based OSI delivery due to favorable blood-to-brain penetration of the drug and additionally by brain targeting route of administration.

This work was aimed at developing liposomal formulations of OSI and a method of its preparation. Prepared liposomes were evaluated in terms of size, loading efficacy, stability, and release profile. Finally, cytotoxicity in NSCLC: A549 with wild-type EGFR gene, PC-9 with a deletion in exon 19 of the EGFR gene, H-1975 bearing mutations T790M and L858R in the EGFR gene, and human lung fibroblasts, MRC-5, was determined.

## 2. Materials and Methods

### 2.1. Materials

Osimertinib (OSI) was purchased from SelleckChem (Selleck Chemicals LLC, Houston, TX, USA), thiazolyl blue tetrazolium bromide (MTT) from Sigma Aldrich (Merck KgA, Darmstadt, Germany). l-α-Phosphatidylcholine 95% (Egg-PC), 1,2-dipalmitoyl-*sn*-glycero-3-phosphocholine (DPPC), 1,2-distearoyl-*sn*-glycero-3-phosphocholine (DSPC), 1,2-distearoyl-*sn*-glycero-3-phosphor-ethanol-amine-*N*-[methoxy(polyethyleneglycol)-2000] sodium salt (PEG2000-DSPE), and cholesterol (chol; ovine wool, >98%), were purchased from Avanti Polar Lipids (Alabaster, Al, USA).

### 2.2. Methods

#### 2.2.1. Preparation of Liposomes

OSI-loaded liposomes and corresponding non-loaded formulations, presented in Table 1, were prepared by thin lipid hydration with drug-loaded passively during hydration or by an ammonium sulfate gradient-assisted loading.

Mixtures of egg-PC (or DPPC or DSPC), cholesterol, and PEG2000-DSPE at molar ratio 55/40/5 were prepared and dissolved in chloroform. For liposomes prepared by thin lipid hydration, OSI at a lipid to drug weight ratio of ca. 20/1 were added to the lipids mixture. Subsequently, the organic solvent was evaporated at 40 °C to form a thin film which was rehydrated with 10 mM PBS, pH 7.4 (thin lipid hydration) or 250 mM ammonium sulfate (remote loading). In all cases, the hydration step was carried out at temperatures above the gel to liquid crystalline phase transition (Tm) of the phospholipids (at 40 °C for egg-PC, at 50 °C for DPPC and 60 °C for DSPC). Liposomes were extruded gradually using polycarbonate membranes 200 nm (5 times) and 100 nm (6 times) at room temperature using an extruder device from Northern Lipids Inc. (Vancouver, BC, Canada).

To obtain ammonium sulfate gradient, four consecutive dialysis exchanges, by using Spectra-Por^®^ Float-A-Lyzer^®^, with a molecular weight cut-off (MWCO) of 8–10 kDa (Spectrum Laboratories, Inc., Piscataway, NJ, USA), against 10% sucrose were used. An ethanol solution of OSI was added to the liposome dispersion after the creation of an ammonium sulfate gradient, during vigorous shaking on an orbital incubation shaker GYROMAXTM 703 (Amerex Instruments Inc., Concord, CA, USA) at 250 rpm for 1 h. The loading was performed at temperatures above the T_m_ of egg-PC (45 °C), DPPC (55 °C), DSPC (65 °C).

Non-entrapped OSI was removed by three consecutive dialysis exchanges: against 10 mM PBS, pH 7.4 for liposomes prepared by thin lipid hydration, or 10% sucrose (1 step), followed by 10 mM PBS, pH 7.4 (2 steps) in case of vesicles loaded with OSI via ammonium sulfate gradient. Subsequently, liposomes were diluted ten times in acetonitrile, and OSI was determined spectrophotometrically by reading absorbance at 308 nm. Encapsulation efficacy (EE, %) was calculated according to Equation (1):

EE (%) = (*C*_M_/*C*_i_) × 100 (1)

where *C*_M_ is the concentration of OSI loaded into liposomes, determined spectrophotometrically, *C*_i_ is the maximum concentration of OSI added during liposome preparation.

#### 2.2.2. Dynamic Light Scattering (DLS)

The mean size and zeta potential of liposomes were determined by DLS (Malvern Zetasizer Nano ZS; Malvern Instruments Ltd., Malvern, UK). Results were obtained in the backscattering mode at a temperature of 25 °C. Each sample was measured multiple times in a consecutive manner, where each analysis consisted of three measurements from which the average size and the polydispersity index (PDI) was calculated.

#### 2.2.3. Stability of Liposomes

For the long-term stability, liposomes were stored in PBS at 4 °C, and particle size and PDI were evaluated at days 0, 60, 90.

#### 2.2.4. In Vitro Release of OSI from Liposomes

In vitro release of OSI from liposomes was performed by the dialysis method in both neutral (pH 7.4) and acidic (pH 6.1) conditions. 10 mM PBS was used as a release medium. 1 mL of liposomal OSI formulations were added to dialysis bags (8 kDa MWCO, Spectra/Por, Spectrum Laboratories, Inc., Piscataway, NJ, USA) and immersed into 40 mL of release medium (*n* = 2) in 50 mL tubes. The tubes were protected from light and shaken at 150 rpm and 37 °C using an orbital incubation shaker GYROMAXTM 703 (Amerex Instruments Inc., Concord, CA, USA). 0.5 mL samples were withdrawn from the release medium and replenished with 0.1% formic acid in ACN at the predetermined time points. Samples were analyzed by high-performance liquid chromatography (HPLC) using an XTerra C18 column, 125 Å, 5 µm, 4.6 mm × 150 mm, (Waters Corporation, Milford, MA, USA) operated at room temperature. The mobile phase consisted of 0.1% formic acid in water/acetonitrile 40:60 (*v:v*), the flow rate was set to 0.5 mL/min, and the OSI signal was detected at 308 nm. The chromatographic apparatus consisted of a Model 1525 pump (Waters Corporation, Milford, MA, USA), a Model 717 Plus auto-injector (Waters Corporation), and a Model 2487 dual-wavelength UV/vis detector (Waters Corporation).

The release kinetics were presented in the form of a ratio of drug released/drug added to dialysis bags against time. The kinetics of OSI release from liposomes was determined using DDSolver software by fitting obtained results to different kinetic models: Higuchi, Krosmeyer-Peppas, Peppas-Sahlin [34].

#### 2.2.5. Cell Culture

Human fibroblasts derived from lungs (MRC-5), non-small-cell lung carcinoma cell lines with wild-type EGFR gene (A549), with a deletion in exon 19 of the EGFR gene (PC-9) and bearing mutant EGFR genes T790M and L858R (H-1975), cell culture media, fetal bovine serum (FBS) and penicillin/streptomycin (P/S) were purchased from the American Type Culture Collection (Manassas, VA, USA). Cells were maintained according to a protocol in the medium supplemented with 10% FBS and 1% P/S: H-1975, PC-9 in Roswell Park Memorial Institute (RPMI) 1640 medium, MRC-5 in Eagle’s Minimum Essential Medium (EMEM), A549 in Kaighn’s Modification of Ham’s F-12 Medium (F-12K).

#### 2.2.6. In Vitro Cellular Cytotoxicity

Cells were seeded onto 96-well plates at a density 5 × 10^3^ cells/well in the culture medium, and allowed to adhere for 24 h at 37 °C. A stock solution of non-loaded drug in dimethyl sulfoxide (DMSO) and liposomal formulations were diluted using cell culture media up to a final drug concentration ranging from 0.001–5 µM. Cells were then treated with serially diluted OSI, OSI liposomal formulations and non-loaded liposomes for 72 h. Non-treated cells served as a control. Cell viability was assessed by MTT assay. Briefly, 100 µL of MTT in cell culture medium (1 mg/mL) was added to each well and incubated for 3 h. Subsequently, the medium was removed, and formazan crystals were dissolved with DMSO. The absorbance was measured as a difference between absorbance measured at a measurement wavelength of 570 nm and a reference wavelength of 650 nm in an Infinite^®^ NanoQuant M200PRO microplate reader (Tecan Group Ltd., Männedorf, Switzerland). Cell viability was determined as a percentage of control cells. A normalized dose-response inhibition curve fitting was performed in GraphPad Prism 8 (GraphPad Software, San Diego, CA, USA), and subsequently, IC_50_ values were calculated.

#### 2.2.7. Cellular Uptake and Localization of Liposomes

Aliquots of fluorescently-labeled liposomes were prepared for each type of studied formulation according to a procedure described above and presented in Table 1. Phospholipid membrane was stained by the addition of 0.2 mol% ammonium salt 1,2-dioleoyl-*sn*-glycero-3-phosphoethanolamine-*N*-(lissamine rhodamine B sulfonyl) with red fluorescence, (Avanti Polar Lipids). Oregon Green^®^ 488 paclitaxel (Thermo Fisher Scientific, Waltham, MA, USA) with green fluorescence was used as a model drug at a final concentration of 1 µM, following the manufacturer protocol. Prior to the visualization by confocal microscopy, MRC-5 and H-1975 cells were plated (10,000 cells/well) in Nunc™ Lab-Tek™ II Chambered Coverglass with a No. 1.5 borosilicate glass bottom, 4-well format plates (Thermo Scientific™), and incubated for 24 h at 37 °C. Fluorescently-labeled formulations were added, and cells were incubated for another hour. Subsequently, the medium was removed, cells were washed twice with PBS and incubated again for 20 min with 5 µM nuclear dye 4′,6-diamidino-2-phenylindole (DAPI, Thermo Fisher Scientific). Cellular internalization of fluorescently-labeled OSI liposomes was analyzed by a confocal microscope (Leica TCS SP8, Leica Microsystems, Wetzlar, Germany).

#### 2.2.8. Statistical Analysis

Statistical analyses were performed with one-way analysis of variance ANOVA followed by Dunnett’s multiple comparison test using GraphPad Prism version 8.0 for Windows. The results were presented as the mean ± SD from three or two independent experiments; *p* values lower than 0.05 were considered statistically significant.

## 3. Results and Discussion

The ultimate goal of the presented study was the development of a liposome-based carrier capable of delivering a novel TKI, osimertinib. TKIs comprise a large group of therapeutic compounds that have revolutionized anticancer therapy, leading to personalized treatment based on molecular profiling [4,5].

The high cargo capacity and the existence of several clinically approved products make nanoparticulate liposomal vesicles ideal candidates for TKIs delivery [19,21,35,36]. The development of optimal liposome-based formulations requires evaluation of a range of parameters that determine performance in vivo, including size, charge, membrane fluidity, particle surface characteristics, and drug loading. It has been shown in numerous preformulation studies that the degree of drug loading, liposomes size, pharmacokinetic properties depends on nature (i.e., physicochemical properties) of drugs and the composition of the liposomes (lipids, lipid/cholesterol ratio, drug/lipid ratio, and charge of the liposomes), as well as the method used for preparation [37].

### 3.1. Preparation Method and Composition

TKIs are mostly hydrophobic, weakly basic molecules. These properties make TKI potential candidates to be carried by liposomes. TKI can be loaded both in the membrane bilayer via passive loading and in the internal aqueous core by applying active loading; both strategies have yielded clinically useful formulations of other drugs and also have been widely explored to prepare liposomal formulations of other TKIs [17,18,21,38]. Here, we examined and compared the properties of liposomes loaded with OSI (a new TKI applied in NSCLC) either by a passive or active method. Characteristics of 12 types of liposomes used in this study, which differ from each other in lipid composition and method used for drug loading is presented in Table 1.

With the passive loading method, the drug is loaded during liposome formation and is incorporated within the lipid membrane.

**Figure 1:**
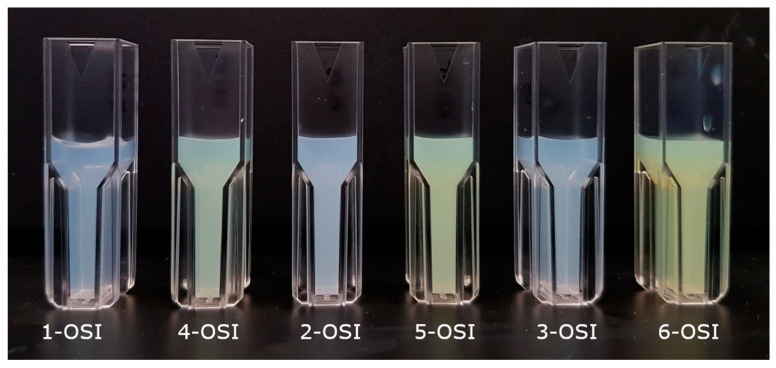
Appearance of liposomal solutions in PBS, pH 7.4 containing liposomes loaded with osimertinib (OSI), is dependent on the drug loading method. Samples loaded with OSI by passive loading are opalescent (1-OSI, 2-OSI, 3-OSI), because the drug is incorporated (solubilized) in the lipid matrix. Samples loaded by ammonium sulfate gradient are yellow (4-OSI, 5-OSI, 6-OSI), which is caused by OSI precipitated in the liposomal aqueous core.

In contrast, with the active loading triggered by ammonium sulfate gradient, a drug is loaded after liposome formation. The resulting OSI-loaded formulations can be distinguished simply by visual observations. As presented in Figure 1, samples loaded with OSI by passive loading are opalescent (1-OSI, 2-OSI, 3-OSI) because the drug is incorporated (solubilized) in the lipid matrix. Samples loaded by ammonium sulfate gradient are yellow (4-OSI, 5-OSI, 6-OSI), due to OSI precipitated in the liposomal aqueous core. Similarly, the market-approved liposomal product of doxorubicin was prepared by a transmembrane ammonium sulfate gradient with doxorubicin precipitated as a sulfate salt inside vesicles [39,40].

In this study, to prepare OSI-liposomes, we used phospholipids and lipid molecules (cholesterol) that have been widely used to formulate liposome-based drug delivery systems, including those clinically approved.

### 3.2. Encapsulation Efficacy

**Figure 2:**
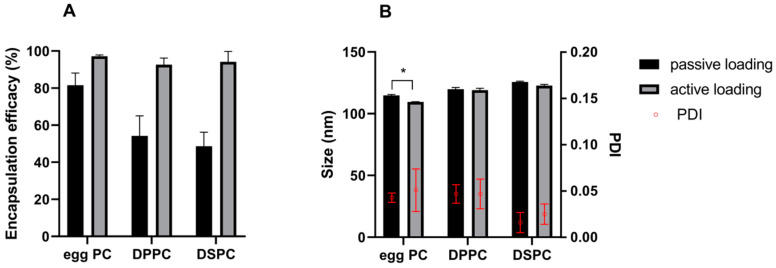
(**A**)—Encapsulation efficacy (%) of osimertinib (OSI) into liposomes composed of different phospholipids (egg-PC, DPPC, DSPC), via passive and active loading method; (**B**)—size and polydispersity index (PDI) of OSI-loaded liposomes. Means ± SD are shown. * *p* < 0.05.

The passive loading is an equilibrium process dependent on parameters, such as the internal and external liposomal volume ratio, drug concentration and solubility in the hydration medium, amount of lipids used to prepare vesicles. The encapsulation efficacy is usually lower compared to remote/active loading [41]. OSI is encapsulated at higher loads, nearly 100% when prepared via active loading, irrespectively of PC type used (Figure 2A). Encapsulation efficacy varies among samples prepared by thin lipid hydration accompanied by passive loading of the drug with the use of different PC types. Figure 2A demonstrates that the highest loading efficacy, in case of samples loaded passively, was observed for liposomes composed of an egg-PC of natural origin, which is unsaturated, asymmetric phospholipid. EE was decreased for saturated, symmetrical DPPC (C16) and further for a long chain (C18), DSPC. Encapsulation efficacy can be affected by many factors involving the preparation method, a type of phospholipid used. Here, we have observed that the loading decreased with an increase in phase transition temperature of the PC, forming the liposomal membrane. Our results are in agreement with research presented by Trummer et al. [17] regarding the loading of gefitinib into liposomal vesicles. Briefly, in the study, they described poor gefitinib incorporation into liposomes when solid, high phase transition lipids such as DSPC where used on the contrary to the more fluid vesicles [17].

### 3.3. Liposomes Size

Liposome size and size distribution are critical attributes of the nanoparticulate formulation. Liposome size contributes to the passive tumor-targeting of nanoparticles via enhanced permeation and retention (EPR) [42]. Therefore the vast majority of liposome-based systems, being used as drug delivery agents, involving clinically approved products, have particles of 80–130 nm. For example, vesicles of liposomal doxorubicin (Doxil^®^) have diameters in the range 80–100 nm, liposomal vincristine (Marqibo^®^)—100 nm, while nanoparticle albumin-bound paclitaxel (Abraxane^®^) has particles size of 130 nm [38,43,44]. Dynamic light scattering studies revealed that tested OSI-loaded liposomes had diameters in the range of 109.6–125.7 nm (Figure 2B). Egg-PC-based formulations seemed smaller than those prepared with DPPC or DSPC. These results were statistically significant for samples prepared by active loading, and for egg-PC and DSPC formulations obtained by passive loading. We did not observe any profound influence of the drug loading method on the liposome size. Statistically significant differences (*p* < 0.05) were found only in the case of formulations containing egg-PC and the liposomes prepared by passive loading had a bigger size (114.8 ± 0.8 nm) than the actively loaded ones (109.6 ± 0.1 nm).

The polydispersity index (PDI) for all studied formulations was below 0.1, pointing out homogenous and monomodal size distribution. The values of PDI of DSPC-based liposomal samples were lower than egg-PC- and DPPC-based formulations. However, the differences were not statistically significant.

### 3.4. Stability

Liposomes represent the example of colloidal systems; therefore, in certain circumstances, the particles in a liposomal dispersion may adhere to one another and form aggregates of successively increasing size, which may settle out under the influence of gravity. The liposomal surface was modified with PEG by incorporating into the liposomal membrane a modified phospholipid, PEG2000-DSPE (5 mol%), to enhance steric stability of developed liposomes and reduce its uptake by cells of the reticuloendothelial system.

**Figure 3:**
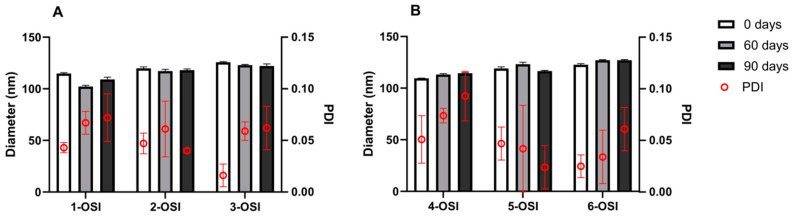
Particle size and PDI stability of OSI-loaded liposomes prepared by passive loading (**A**) or active loading (**B**), during 3-month storage in PBS, pH 7.4 at 4 °C. DLS measurements were performed on day 0, 60, 90 following preparation (*n* = 3). Means ± SD are shown.

Physical stability of the liposomal dispersion is usually monitored by controlling the size of the particles over the storage time in the medium of choice (buffer or albumin containing buffer solution). In the presented study, a 90-day physical stability test of the OSI-loaded liposomes stored at 4 °C in 10 mM PBS (pH 7.4) was conducted. DLS measurements were performed on day 0, 60, 90, following preparation (Figure 3), and no statistical differences were found in the particle size over time.

Zeta potential is a standard analytical measurement for nanoparticle surface characterization. It is a parameter often used to monitor the stability of colloidal dispersion. The zeta potential of ‘naked’ liposomes (with no PEG present on the liposome surface) was ca. −40 mV (data not shown). The zeta potential of the studied OSI-liposomes (1-OSI–6-OSI) and drug-free liposomes (1-NL–6-NL) was measured by DLS. The determined values were in the range of −10.2–−11.9 mV. These data correspond to the zeta potential reported for liposomes containing similar content (2–5 mol%) of PEG derivatized phospholipid previously reported [45,46]. The data also agree with the finding that the PEG chains covering the liposome surface reduce the absolute value of zeta potential [45,46]. The zeta potential was shown to get lower with increasing concentration of PEGylated phospholipid and eventually reached a plateau around −5 mV [46]. That decrease in the absolute value of zeta potential can be attributed to the following mechanisms: (i) the slipping plane being moved further away from the liposome surface and (ii) the drag caused by the presence of the PEG chains on the liposome surface reducing the mobility of the liposomes (and hence the zeta potential) [46].

### 3.5. In Vitro Drug Release

The mechanism of drug release from liposomes is based on passive drug permeation and diffusion. Drug release from liposomal carriers is a complex process affected by the physicochemical properties of the liposome and the physical state of the encapsulated drug, as well as external factors such as the release medium selection, temperature and pH. The physicochemical properties that determine drug release from liposomes include drug permeability, drug ionization constant, drug binding behavior with the lipid bilayer, self-association of the drug, and the presence of intraliposomal precipitate [47].

The OSI release from liposomes was studied in vitro by a dialysis method. The effect of preparation method and vesicle composition on the release profile was evaluated. The influence of neutral (7.4) and acidic (6.1) pH on OSI release from liposomes was also studied regarding different pH conditions in the extracellular environment of normal and cancer cells. Acidification of the extracellular milieu and concomitant intracellular alkalization of the cytoplasm are well-known hallmarks of cancer [48,49], which may affect the release profile of the drug, mostly weak acids and bases.

**Figure 4:**
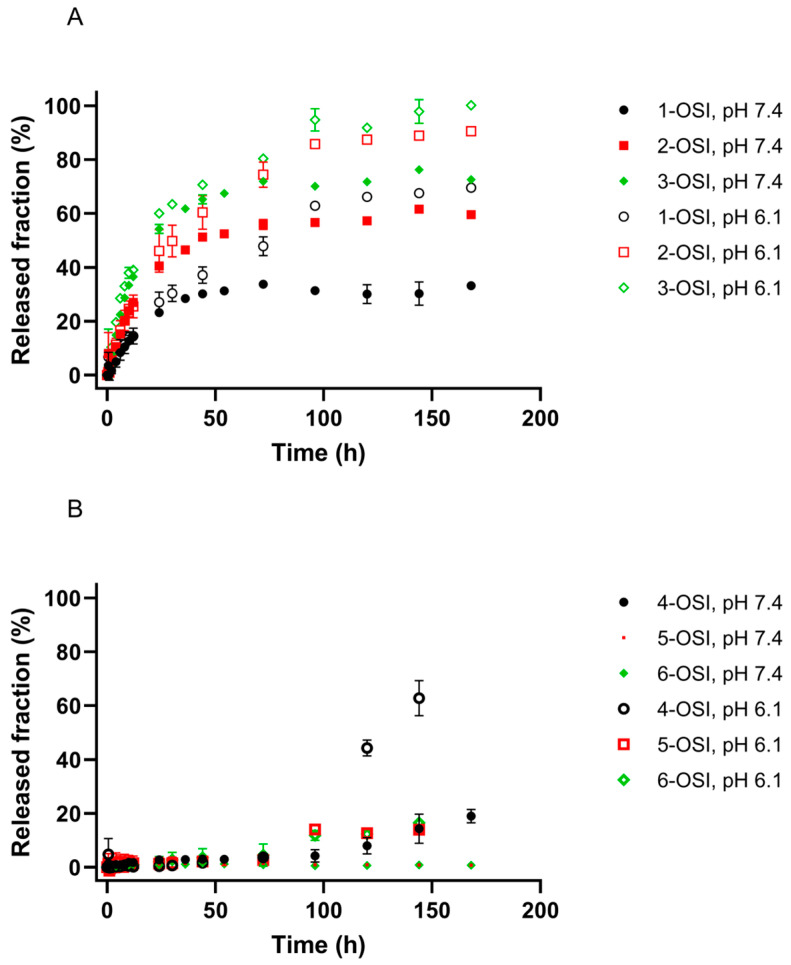
Cumulative release profiles of osimertinib (OSI) from liposomes composed of egg-PC (black circles), DPPC (red squares), DSPC (green diamonds) prepared by passive loading (**A**) or active loading (**B**). 10 mM PBS was used as a release medium with pH adjusted to neutral (7.4) or acidic (6.1). Means ± SD are shown.

The experimental release profiles, presented in Figure 4, show that OSI, being located within different liposomal compartments and in a different molecular form, behaves differently. As mentioned in the paragraph Preparation method and composition, OSI loaded passively is incorporated into the lipid membrane. In that compartment of the liposome, OSI is present in the non-ionized form, which was quickly released from the vesicles when PBS at both pH values was used (Figure 4A). In the case of gradient-loaded liposomes, OSI was encapsulated in the form of precipitated salt (sulfate). Despite the different pH conditions applied, a standard release-testing method using phosphate-buffered saline is not sufficient (see Figure 4B), because the drug release is not dominated by simple diffusion but by a gradient-related diffusion [50,51].

The time (T_50_) at which approximately 50% of OSI was released from passively-loaded liposomes was estimated and shown in Table 2. It can be noted that T_50_ decreased when the PBS buffer at pH 6.1 was used as a release medium. This can be explained by increased solubility of OSI (a weak base) in an acidic environment, which enabled quicker diffusion of drug molecules. Furthermore, the release profiles in Figure 4A, showed that an increase in drug release rate is not only pH-dependent but also depends on the character of the dominant phospholipid.

The highest release rate was observed for DSPC, which simultaneously is characterized by the highest T_m_. Due to the lower transition temperature of egg-PC (25 °C) in comparison to DPPC (49 °C) and DSPC (55 °C), egg-PC/cholesterol liposomes are more fluid than the DPPC/cholesterol or DSPC/cholesterol liposomes at 37 °C [39]. Usually, more fluid liposomal formulations are more “leaky” and release an incorporated drug at a higher rate. In the presented study, however, the drug release rate increased when the saturated phospholipids were used (egg-PC vs. DPPC) and with the length of the lipid carbon chains (DPPC vs. DSPC). Both higher OSI encapsulation efficacy and slower release rate from egg-PC/cholesterol-based liposomes may point out that there some nonpolar regions (“pockets”) of the membrane which interact with OSI stronger than those formed in the liposome membrane based on DPPC or DSPC.

### 3.6. Mathematical Modeling of OSI Release from Liposomes

To better understand the release mechanism underlying OSI release from evaluated formulations, data were fitted to the following mathematical models: Higuchi, Peppas-Sahlin, Korsmeyer-Peppas, using DDSolver software [34].

Mathematical models are an essential tool to design pharmaceutical formulations, evaluate drug release processes in vitro and in vivo and, in general, come up with the optimal design for new systems [53]. In general, when a hydrophilic drug is incorporated in a matrix, the release occurs easily by diffusion, compared to a hydrophobic or less water-soluble drug. The hydrophobic drug release is typically associated with swelling and/or matrix erosion. The mechanism by which the drug release is governed can be determined by statistical analysis of the first 60% of all release curves and is based on the higher *R*^2^ and lower Akaike information criterion (AIC) [52]. As shown in Table 2, *R*^2^ is the highest while AIC is the lowest for the Peppas-Sahlin model in almost all samples, apart from 1-OSI at pH = 6.1. The Peppas-Sahlin model is a release kinetics model that assumes two contribution mechanisms, diffusional and relaxational, in an anomalous drug release process. According to Korsmeyer-Peppas release exponent value *n*, and Peppas-Sahlin contribution coefficient *m*, drug dissolution in combination with diffusion and liposome relaxation is considered as the mechanisms for OSI release from the liposomal matrix.

### 3.7. Cellular Uptake and Localization of Liposomes

**Figure 5:**
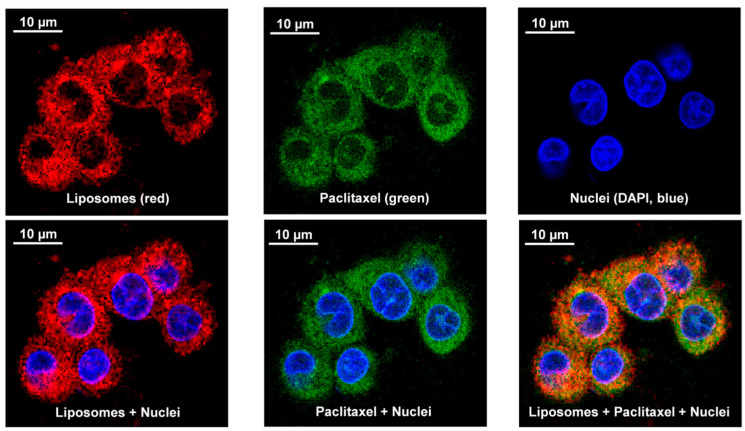
Cellular Internalization of Liposomes (Red Fluorescence) Containing Paclitaxel (Green Fluorescence) by Confocal Microscope (Leica TCS SP8, Leica Microsystems, Wetzlar, Germany). Human H1975 non-small lung cancer cells were incubated with liposomes (Rhodamine, red fluorescence) containing paclitaxel (Oregon Green, green fluorescence). Nuclei were stained with DAPI (blue fluorescence). Superimposition of red and green colors gives yellow color. Representative images are shown.

**Figure 6:**
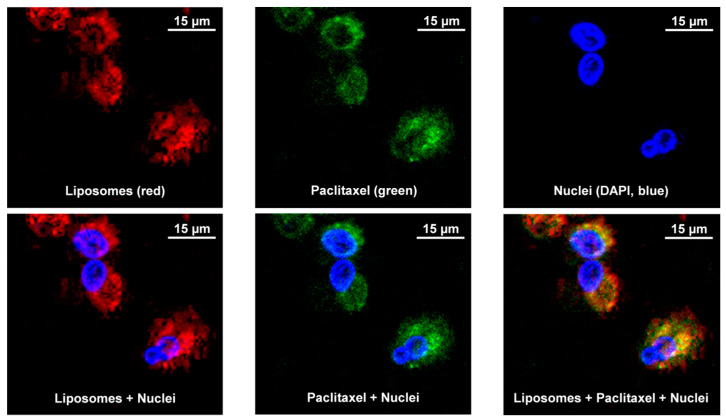
Cellular Internalization of Liposomes (Red Fluorescence) Containing Paclitaxel (Green Fluorescence) by Confocal Microscope (Leica TCS SP8, Leica Microsystems, Wetzlar, Germany). Human MRC-5 normal lung cells were incubated with liposomes (Rhodamine, red fluorescence) containing paclitaxel (Oregon Green, green fluorescence). Nuclei were stained with DAPI (blue fluorescence). Superimposition of red and green colors gives yellow color. Representative images are shown.

In order to show that liposomes with incorporated drug can penetrate into cellular cytoplasm and release the payload, we labeled liposomes (1-OSI formulation) with rhodamine (red fluorescence) and used commercially available paclitaxel labeled with Oregon Green (green fluorescence) and incubated with human H-1975 EGRF mutation-positive non-small lung cancer cells and MRC-5 diploid human cell culture line composed of fibroblasts derived from lungs. In addition, cell nuclei were stained with DAPI nuclear dye (blue fluorescence). It is known that liposomes are quenching fluorescence of compounds incorporated into liposomes [54]. Thus, the fluorescence of labeled drug (paclitaxel) is quenched inside the liposomes. Therefore, a presence of green fluorescence inside the cellular cytoplasm documents the release of the drug from the liposomes and its intracellular localization. It can be seen that liposomes with the drug penetrated cancerous and normal lung cells, localized predominately in the cytoplasm and release the drug into the cytoplasm. The representative images shown in Figure 5 and Figure 6 demonstrate that liposomes are successfully internalized into the both investigated cells and released the drug.

Generally, liposome particles are taken up by endocytotic processes and eventually reach the lysosome [55,56]. In the lysosome, liposomes and their encapsulated cargo are exposed to acidic conditions and the risk of being degraded by lysosomal enzymes [57]. For example, liposomal doxorubicin Doxil^®^, was shown to utilize caveolae-mediated endocytosis to internalize into epithelial cancer cells in a study by Sahay et al. [58]. Following internalization, DOXIL^®^ nanoparticles accumulated in lysosomes, where the doxorubicin was released [58]. Based on the above reports and the known pathway of liposome internalization via endocytosis, it can be assumed that liposomes reach lysosomes where acidic pH conditions and enzymatic degradation of the carrier enables drug release and support solubilization (due to protonation) of OSI molecules. As we noticed in the in vitro release study, the OSI release rate, well seen in case of passively loaded liposomes, increases with the decrease of pH. Furthermore, we may also assume that the degradation of liposomes in the lysosomal compartment may trigger and accelerate the release of OSI from actively loaded liposomes.

### 3.8. Cytotoxicity

The cytotoxic activity of liposomal OSI was evaluated in vitro across MRC-5 cells, which are human fibroblasts derived from lungs and H-1975 cells, which is non-small-cell lung carcinoma cell line bearing mutant EGFR gene (T790M and L858R). For these studies, the incubation time of the formulation with the cells was 72 h with OSI at a concentration of 0.0001–5 µM, based on previous literature reports [59,60].

**Figure 7:**
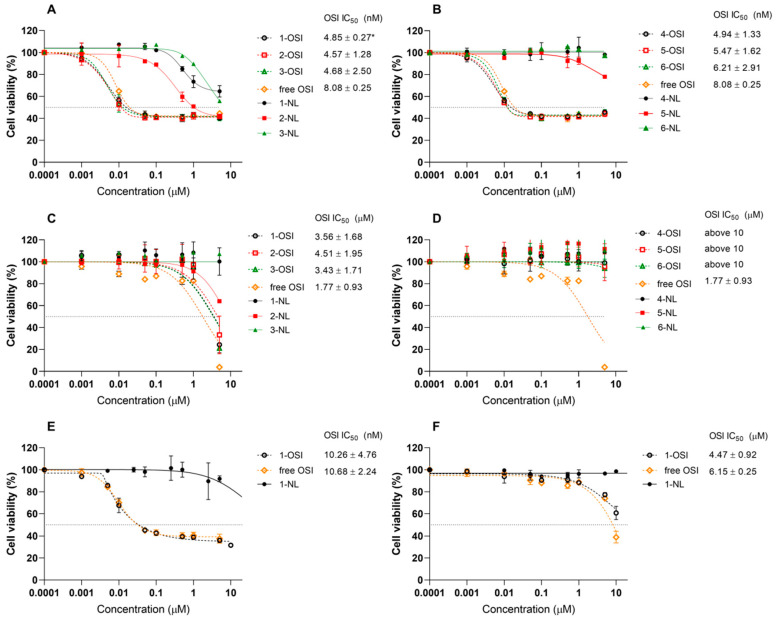
In vitro cytotoxicity and IC_50_ of free OSI, liposomes loaded with OSI passively (1-, 2-, 3-OSI—(**A**,**C**,**E**,**F**)) and actively (4-, 5-, 6-OSI—(**B**,**D**)) in H-1975 (**A**,**B**), MRC-5 cells (**C**,**D**), PC-9 (**E**), A549 (**F**). To examine the possible cytotoxicity of non-loaded (NL) liposomes, for the cytotoxicity experiments, empty liposomes 1-NL–6-NL were diluted by the same dilution factor as corresponding OSI-loaded formulations. For each group *n* = 2 or *n* = 3. Means ± SD are shown. * *p* < 0.05 when compared to free non-incorporated drug.

In H-1975 cells, all liposomal formulations of OSI exhibited lower IC_50_ (4.57–6.21 nM) compared to free OSI (8.08 nM) in MTT assay (Figure 7). However, the IC_50_ of only 1-OSI was significantly lower in comparison to the non-incorporated drug (*p* < 0.05). As shown in Figure 7, MRC-5 cells, which do not overexpress EGFR, do not respond to OSI treatment, either a free drug or liposomal form, at concentrations lower than 1 µM. An MTT assay was conducted on liposomes without drug incorporated, by using the same dilutions of non-loaded liposomes as used for OSI-loaded samples to examine the possible bioactivity of non-loaded liposomes themselves against H-1975 and MRC-5. The results show that 1-NL–6-NL liposomes only demonstrated a minor impact on the proliferation of the H-1975 and MRC-5 cells at extremely high lipid concentration with co-incubation times as long as 72 h.

Considering the physicochemical properties and biological activity, we have found egg-PC liposomes as the most promising formulation for further development. Therefore, we additionally tested the cytotoxicity of 1-OSI, 1-NL formulations, and free OSI in other NSCLC, PC-9 cell line with a deletion in exon 19 of the EGFR gene which is sensitive to OSI [61], and A549 line with wild type EGFR gene which is known to be resistant to OSI treatment [62]. As shown in Figure 7E,F, cytotoxicity profiles determined for OSI and its liposomal form (IC_50_ at ca. 10 nM) in PC-9 cells are similar to those plotted for the same formulations in H-1975 (Figure 7A). Egg-PC-based liposomal OSI below 5 µM did not affect the viability of A549.

## 4. Conclusions

In the study, we proposed a liposome-based delivery system for novel TKI, osimertinib, which is used for the personalized treatment of NSCLC with EGFR mutations. We showed that OSI could be loaded into liposomes passively by hydration method or actively by gradient-loading technique. The above methods led to OSI incorporation in the liposomal membrane and in the aqueous core, respectively. The obtained vesicles did not differ much in terms of size, PDI, zeta potential, and stability. Since drug substance was located in different compartments of the liposome, different entrapment efficacy and OSI release profiles were achieved.

Based on the presented results, mostly encapsulation efficacy, in vitro drug release and cytotoxicity, we identified liposomes composed of egg-PC as the most suitable carrier for OSI that can be considered for further development. Above all, OSI loaded into liposomes composed of egg-PC showed higher antitumor efficacy against cancer cells with EGFR mutations than free OSI and did not affect the viability of normal lung fibroblasts. Apart from improved drug performance, the choice of egg-PC, which is a phospholipid of natural origin, can be considered advantageous for further development. Natural phospholipids are more sustainable products derived from abundantly available raw material sources and are preferred compared to synthetic phospholipids [63].

In the future perspective, with a formulation developed, we can continue our studies on liposomal OSI and consider various routes of administration (intravenous, inhalation). All the more important, there is a clinical need for novel EGFR-TKIs with improved efficacy against brain lesions. The ability of OSI to penetrate the blood-brain barrier and the potential usefulness in the treatment of CNS metastases or leptomeningeal disease (related to NSCLC), supported by effective drug carriers and delivery route, may bring this concept to success.

## Figures and Tables

**Table 1:** Characteristics of 12 types of liposomes used in this study, which differ from each other in lipid composition and method used for drug loading.

Formulation	Liposome Components	Initial Molar Ratio	Size (nm)	PDI	Zeta Potential (mV)	EE (%)
**Thin Lipid Hydration—Passive Loading**						
1−NL	Egg−PC/chol/PEG2000−DSPE	55/40/5	108.7 ± 1.3	0.101	−10.9 ± 0.3	
1−OSI	Egg−PC/chol/PEG2000−DSPE/OSI	55/40/5/7	114.8 ± 0.8	0.043	−11.4 ± 0.8	81.5 ± 6.6
2−NL	DPPC/chol/PEG2000−DSPE	55/40/5	122.0 ± 1.0	0.036	−11.5 ± 0.0	
2−OSI	DPPC/chol/PEG2000−DSPE/OSI	55/40/5/7	119.8 ± 1.4	0.047	−10.3 ± 0.7	54.2 ±10.8
3−NL	DSPC/chol/PEG2000−DSPE	55/40/5	124.4 ± 2.0	0.049	−11.6 ± 1.4	
3−OSI	DSPC/chol/PEG2000−DSPE/OSI	55/40/5/7	125.7 ± 0.6	0.016	−10.4 ± 0.5	48.6 ± 7.6
**Thin Lipid Hydration—Ammonium Sulfate Gradient−Assisted Loading**						
4−NL	Egg−PC/chol/PEG2000−DSPE	55/40/5	110.9 ± 1.0	0.042	−10.3 ± 0.2	
4−OSI	Egg−PC/chol/PEG2000−DSPE/OSI	55/40/5/7	109.6 ± 0.1	0.051	−10.2 ± 0.9	97.2 ± 0.7
5−NL	DPPC/chol/PEG2000−DSPE	55/40/5	124.0 ± 0.6	0.030	−10.2 ± 0.9	
5−OSI	DPPC/chol/PEG2000−DSPE/OSI	55/40/5/7	119.1 ± 1.4	0.047	−11.9 ± 1.5	92.6 ± 3.6
6−NL	DSPC/chol/PEG2000−DSPE	55/40/5	125.5 ± 0.6	0.097	−11.5 ± 1.1	
6−OSI	DSPC/chol/PEG2000−DSPE/OSI	55/40/5/7	122.7 ± 1.0	0.025	−11.3 ± 1.3	94.2 ± 5.6

**Table 2:** Parameters obtained by fitting the in vitro release data of OSI from liposomal formulations to three mathematical models.

Model	Parameter	Formulation					
		1−OSI	2−OSI	3−OSI	1−OSI	2−OSI	3−OSI
		pH 7.4			pH 6.1		
*Higuchi*; $\frac{M_{t}}{M_{\infty }}=k_{H}t^{1/2}$							
	*k_H_*	3.365	5.816	10.179	5.595	8.141	11.423
	*R* ^2^	0.805	0.882	0.963	0.955	0.942	0.953
	AIC	116.350	126.699	57.458	71.072	61.275	53.025
	T_50_ (h)	220.772	73.910	24.130	79.865	37.723	19.161
*Korsmeyer−Peppas*; $\frac{M_{t}}{M_{\infty }}=k_{KP}t^{n}$							
	*k_KP_*	6.767	10.490	7.805	2.921	5.126	8.030
	*n*	0.342	0.367	0.593	0.665	0.650	0.644
	*R* ^2^	0.886	0.935	0.978	**0.990**	0.980	0.982
	AIC	108.714	117.946	53.791	53.748	51.578	45.218
	T_50_ (h)	346.892	70.771	22.899	71.364	33.181	17.129
*Peppas−Sahlin*; $\frac{M_{t}}{M_{\infty }}=k_{1}t^{m}+\;k_{2}t^{2m}$							
	*k*_1_	3.664	6.200	5.244	2.313	−21.099	−33.843
	*k*_2_	−0.095	−0.157	−0.111	1.209	22.926	38.891
	*m*	0.641	0.624	0.865	0.401	0.200	0.176
	*R* ^2^	**0.974**	**0.985**	**0.989**	0.988	**0.990**	**0.994**
	AIC	82.091	91.581	45.395	55.059	42.983	33.540
	T_50_ (h)	None Calc	48.988	19.872	71.670	32.513	17.074

$M_{\infty }$ is the amount of drug at the equilibrium state (sometimes very close to the amount of drug contained in the dosage form at the beginning of the release process); $M_{t}$ is the amount of drug released over time *t*; $k_{H},$ $k_{KP}$, $k_{1,\;}k_{2}$—are the release constant of Higuchi, Korsmeyer-Peppas and Peppas-Sahlin, and are constants of incorporation of structural modifications and geometrical characteristics of the system; n, m is the exponent of release, related to the drug release mechanism in function of time *t*; *R*—correlation coefficient; AIC—Akaike information criterion; T_50_—time at which 50% of a drug is released [52].
